# Supporting Those Who Go to Fight Ebola

**DOI:** 10.1371/journal.pmed.1001781

**Published:** 2015-01-26

**Authors:** Michelle M. Mello, Maria W. Merritt, Scott D. Halpern

**Affiliations:** 1 Stanford Law School, Stanford, California, United States of America; 2 Department of Health Research and Policy, Stanford University School of Medicine, Stanford, California, United States of America; 3 Johns Hopkins Berman Institute of Bioethics, Baltimore, Maryland, United States of America; 4 Department of International Health, Johns Hopkins Bloomberg School of Public Health, Baltimore, Maryland, United States of America; 5 Perelman School of Medicine, University of Pennsylvania, Philadelphia, Pennsylvania, United States of America

The Ebola epidemic is testing virtually every aspect of the public health and health care systems in the United States, including health care institutions’ public service commitments. Although the number of cases in the US remains very small, an extraordinary amount of public and hospital resources have been devoted to preparing for new cases domestically [1–4]. In contrast, although US hospital and medical professional organizations have called for an “enhanced focus” on containing Ebola in West Africa [5], there is a striking absence of public commitments on the part of US health care institutions to contribute to the containment effort.

By quickly mobilizing qualified health care professionals (HCPs) to work in Guinea, Liberia, and Sierra Leone, US hospitals could not only meet the needs of desperate patients, but could contain Ebola at its source, averting global risk [6]. Yet, US institutions’ response to the West African epidemic has been muted thus far. Reports indicate that many institutions—even those with a tradition of sending personnel to respond to other humanitarian crises—have asked their HCPs to stay home this time [7–9].

Although some academic medical centers (AMCs) in the US have invoked their usual policy that the university will support overseas work with services such as emergency travel assistance, others have specified that staff who serve in Ebola-affected regions do so in their personal capacity, not as employees. Still others have strongly cautioned against serving, prohibited official travel to affected regions, required staff to take vacation time or unpaid leave for 21 days following repatriation before returning to work, and made clear that the university will not assist if the HCP falls ill.

The concerns that may motivate hospitals to discourage volunteers do not outweigh the countervailing considerations. At a minimum, institutions ought not to impede service; ideally, they would promote it.

## Arguments for Facilitating Service

There are several reasons why institutions should facilitate HCPs’ service in Ebola-affected regions. First and most obviously, facilitating the deployment of medical personnel to affected regions in the near future could make the difference between turning the tide of the epidemic and forfeiting the opportunity to avert regional and even global catastrophe [10]. Many have argued persuasively that higher-income countries have a moral obligation to render humanitarian assistance to affected African nations because it can be done at relatively modest cost and because of basic considerations of global justice [11]. But intervening to stop the spread of Ebola is also a vital way to protect the rest of the world. Hospitals ought to consider the role of HCPs’ service in the future protection of their own patients, workers, and communities [6].

Another reason to support service in Africa stems from professional duties to treat. HCPs may be motivated to serve in Ebola-affected regions for different kinds of reasons. They may consider such service an outgrowth of their professional duty to care for the sick, a perception supported by professional ethical guidelines [12,13]. Some may be called to this crisis by its extraordinary scope and urgency. These motivations are not merely matters of personal conscience; they exemplify core values of the health care and public health professions that institutions and society should encourage.

Without institutional support, it is hard, if not impossible, for HCPs to answer the call for service overseas. Furthermore, HCPs should not have to shoulder avoidable risk. The value of reciprocity counsels that those who step forward must be assured of the best medical treatment and maximal respect for their rights [14,15], including the right to return to work in the US with the least possible infringement on their liberty commensurate with ensuring public safety. Providing pay, psychological support, assistance accessing care in the US, and other services all minimize the burden [16].

A final set of considerations applies primarily to AMCs. As institutions of higher learning that receive public and private funds to generate publicly actionable knowledge and to train domestic and global health providers of tomorrow, AMCs are engaged in explicitly outward-facing endeavors. American AMCs not only receive tax exemptions for such public-service endeavors but also receive endowments and other forms of private support, thanks to their branding themselves as institutions with external missions. Such benefits are typically well deserved and serve vital purposes here and abroad, but also carry the responsibility to live up to the advertised goals. Supporting HCPs in serving in Africa is also a way of modeling the medical profession’s ideal behavior to trainees.

## Arguments for Restricting Service

Institutions in the US that have backed away from supporting HCP service in West Africa may have three concerns. First, some AMCs may worry about whether they can adequately protect their employees overseas. Uncertainty surrounds the continued availability of medical evacuation, and there may also be concerns that violence and civil unrest could break out in Ebola-affected countries [7]. Such problems often motivate universities to issue travel advisories or prohibitions. What makes the current situation so challenging, by contrast with the typical scenario of elevated risk, is that both sides of the risk–benefit calculus are magnified. On the risk side, the potential harms to travelers and the surrounding uncertainties are unusually daunting, particularly because of shortcomings in the local infrastructure of affected regions. On the benefit side, whereas university travel is ordinarily conducted for scholarly and teaching purposes, the Ebola epidemic involves rendering an urgently needed public health service that HCPs are uniquely able to provide. These potential benefits are of exceptional magnitude and global in their reach. To minimize the risks and maximize the benefits of HCPs’ service, AMCs and other institutions should require that HCPs who volunteer to serve do so only through established and qualified organizations, and help HCPs to inform themselves fully of all residual risks and uncertainties.

Second, institutions are likely concerned about exposing local patients, staff, and community members to a risk of Ebola infection by returning clinicians. To be sure, health care institutions have ethical and legal obligations to protect these groups. Transmission risks can be minimized by requiring clinical staff to register their travel and prospectively commit to complying with CDC guidelines [17] on their return. Because risks to others are manageable, they should not dominate the discourse about supporting service.

A third concern may be that the protracted absence of key personnel could create operational challenges for hospitals, potentially jeopardizing their ability to serve their patients. Service in Ebola-affected regions may involve a longer deployment than typically occurs in humanitarian disasters, as well as several days of training and perhaps several weeks of remaining away from the workplace after returning. Arranging clinical coverage may be costly and not suffice in the short run to guarantee standard timeliness and staffing of care. Conceivably, nurses might need to care for more patients each than is normal, and continuity of physician care might temporarily worsen. Institutions may also incur costs arranging for the medical evacuation, care, and sick leave of HCPs who develop Ebola symptoms, and in screening returning HCPs to clear them to return to work.

There is a point at which such costs could become so burdensome that a department chair or other hospital leader might reasonably deem it appropriate to limit the number of HCPs who could be absent at once. Minor decrements in the quality of domestic care could be tolerated by health care institutions and patients given the circumstances, but substantial limitations may be more difficult to justify. However, presently, the number of HCPs willing, able, and qualified to go is small. Thus, for many, if not most, hospitals, the burdens encountered would be minor, comparable to those routinely assumed in providing HCPs with family and medical leave.

## Conclusion

Given the opportunities for HCPs to care for patients in desperate need and help avert global harm, the consistency of such service with HCPs’ professional ethics and AMCs’ missions, and institutions’ ability to manage risks attributable to HCPs’ temporary absence and return to work, health care institutions should routinely support willing and qualified HCPs’ service in West Africa. At a minimum, institutions should not impede employees from fulfilling their perceived professional duties to help the sick and, thereby, to do their part in responding to a global public health emergency. This means refraining from adverse action against those who choose to travel, arranging for others to provide the services the travelers normally render, and not imposing restrictions that exceed CDC recommendations for returning travelers.

Ideally, institutions would go further and actively promote HCPs’ service by enabling them to go as employees and preserving the full net of support and protection this status confers in the US, including travel insurance, worker’s compensation coverage, and pay. Such institutions would also assume liability for harms to third parties, although these situations would likely be rare.

Finally, institutions could fulfill their ethical responsibilities to contribute to the fight against Ebola in different ways. Some hospitals might step forward as primary centers of care for Ebola patients domestically while others focus on facilitating HCPs services’ abroad. However institutions choose to address these responsibilities, it is heartening that there are HCPs who wish to provide care in West Africa. This heroism is remarkable and reflects a deep humanitarian instinct. It calls for validation, not discouragement, by health care institutions.
